# A comparative review of viral entry and attachment during large and giant dsDNA virus infections

**DOI:** 10.1007/s00705-017-3497-8

**Published:** 2017-09-02

**Authors:** Haitham Sobhy

**Affiliations:** Department of Molecular Biology, Umeå University, 901 87 Umeå, Sweden

## Abstract

Viruses enter host cells via several mechanisms, including endocytosis, macropinocytosis, and phagocytosis. They can also fuse at the plasma membrane and can spread within the host via cell-to-cell fusion or syncytia. The mechanism used by a given viral strain depends on its external topology and proteome and the type of cell being entered. This comparative review discusses the cellular attachment receptors and entry pathways of dsDNA viruses belonging to the families *Adenoviridae*, *Baculoviridae*, *Herpesviridae* and nucleocytoplasmic large DNA viruses (NCLDVs) belonging to the families *Ascoviridae*, *Asfarviridae*, *Iridoviridae*, *Phycodnaviridae*, and *Poxviridae*, and giant viruses belonging to the families *Mimiviridae* and *Marseilleviridae* as well as the proposed families *Pandoraviridae* and *Pithoviridae*. Although these viruses have several common features (e.g., topology, replication and protein sequence similarities) they utilize different entry pathways to infect wide-range of hosts, including humans, other mammals, invertebrates, fish, protozoa and algae. Similarities and differences between the entry methods used by these virus families are highlighted, with particular emphasis on viral topology and proteins that mediate viral attachment and entry. Cell types that are frequently used to study viral entry are also reviewed, along with other factors that affect virus-host cell interactions.

## Introduction

**Fig. 1:**
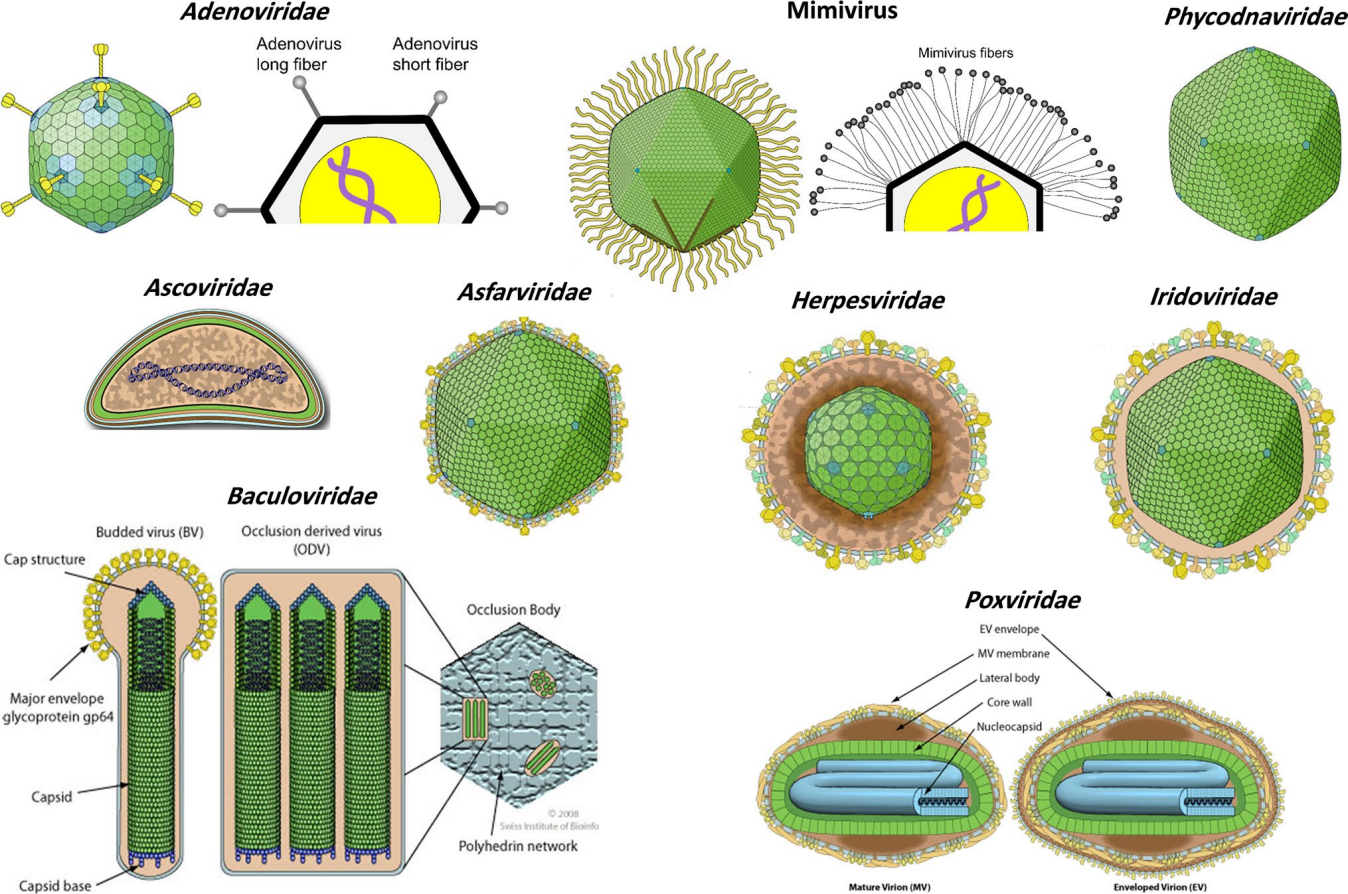
The different virion topologies of the 12 dsDNA large and giant virus families. Image adapted from ViralZone (http://viralzone.expasy.org/) [10]. Schematic representation of the different shapes of adenovirus and mimivirus fibers

Viruses utilize several mechanisms to enter host cells. This review focuses on the relationships between the external topology of the virions and their entry mechanisms in different cell types, as well as the roles of cellular receptors and viral attachment factors. Ten viral families are discussed, including *Adenoviridae*, *Baculoviridae*, *Herpesviridae*, and nucleocytoplasmic large DNA viruses (NCLDVs). The NCLDVs include large and giant viruses characterized by their large virions and genomes, and can be classified into several distinct families: *Ascoviridae*, *Asfarviridae*, *Iridoviridae*, *Mimiviridae*, *Marseilleviridae*, *Phycodnaviridae* and *Poxviridae*. They also include members of the proposed families *Pandoraviridae* and *Pithoviridae* as well as the recently isolated molivirus and faustovirus [1–4]. They replicate completely or partially in the cytoplasm and are larger than other viruses. They may also have several common traits, including similarities in their protein sequences and topological features. Figure 1 shows the external topology of each viral family. They might be evolutionary related and share a common ancestor [5, 6]. It has been proposed that the NCLDVs be classified into one order, named “*Megavirales*” [7], whereas, herpesviruses belong to the order *Herpesvirales*. Generally, mimiviruses and phycodnaviruses are closely related to pandoraviruses and moliviruses, whereas pithoviruses are related to marseilleviruses, iridoviruses and ascoviruses, and faustovirus are closely related to asfarviruses, [1–4, 8, 9].

## Virus attachment and receptors

**Fig. 2:**
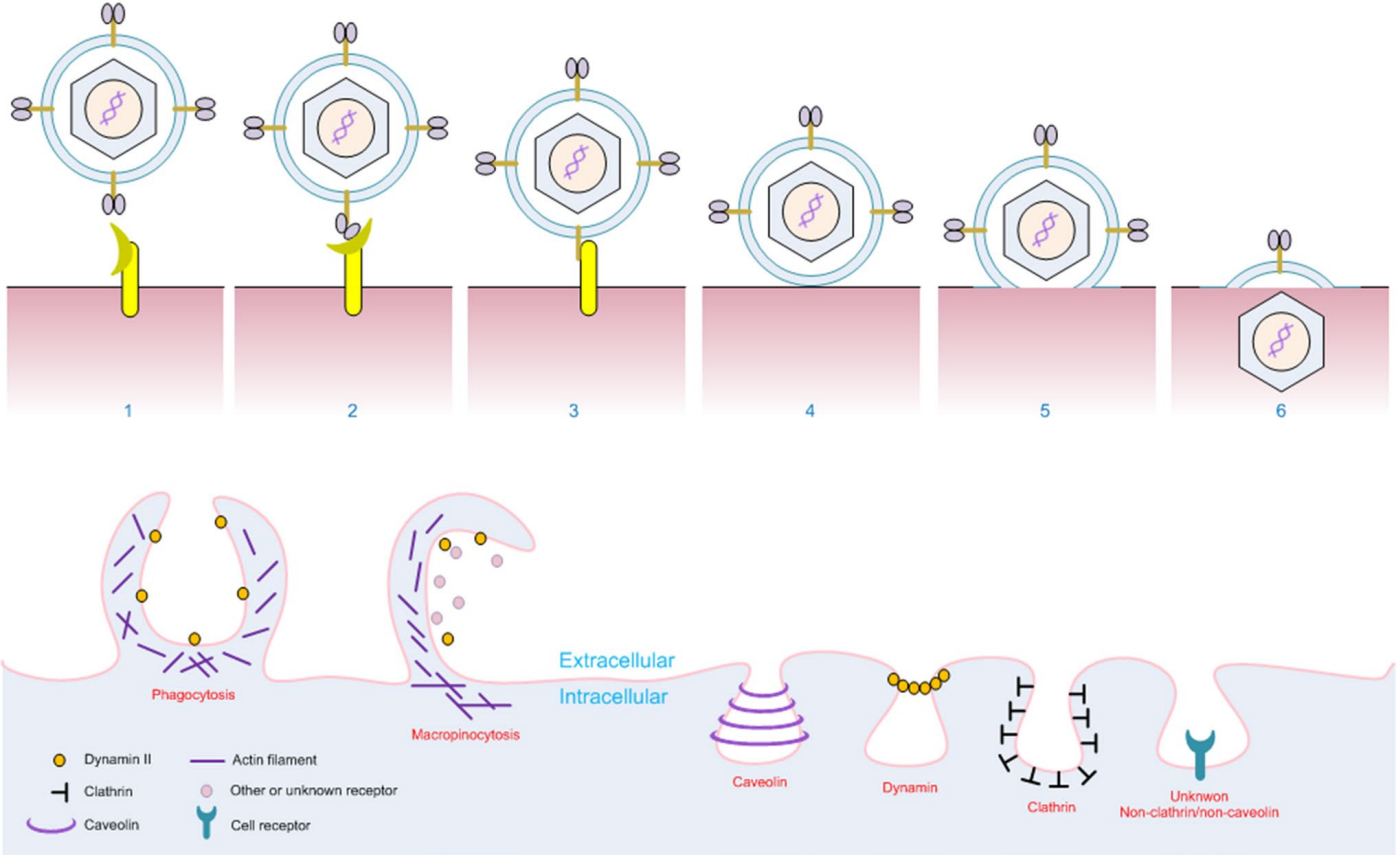
Schematic representation of viral attachment and fusion (upper panel) and entry mechanisms (lower panel)

Viruses attach to proteins known as cellular receptors or attachment factors on the surface of the host cell [11, 12]. In addition, certain membrane lipids and glycans may be necessary for viral entry. These factors stabilize the virus on the cell surface and allow it to circumvent the cell’s barriers to entry. High-affinity interactions between viral proteins and cellular receptors drive conformational changes in the proteins’ structures that activate signaling cascades and destabilize the plasma membrane, leading to pore formation and internalization of the virus as shown in Figure 2a [13]. These interactions can be initiated by specific motifs or domains in both viral and host proteins. Notable viral protein motifs that facilitate entry by binding to cellular counterparts include the integrin-binding (RGD), endocytosis (PPxY and Yxx[FILV]), and clathrin endocytosis (PWxxW) motifs, where “x” denotes any residue [14]. It is worth noting that a receptor could be accompanied by an additional co-receptor that triggers a particular entry pathway or stabilizes the virus at plasma membrane.

## General mechanisms of virus entry

Cells can internalize viruses by endocytosis, as reviewed elsewhere [11–13, 15–17] and depicted in Figure 2. Alternatively, the virus may fuse with the cell membrane. Several factors determine which entry mechanism will be active, including the cell type and the cellular receptors it displays. Aspects of the virus’ external topology, such as the presence of surface protrusions or glycoproteins, may also affect the entry process. Viruses enter host cells via one of three major pathways:

**(A) Fusion**: Viral proteins promote the fusion of the virion with the plasma membrane, which then form a pore, and the virion becomes uncoated. Its genomic cargo is then transferred into the cytoplasm [12, 13, 18–21]. The proteins involved in fusion, so-called fusogens, can be divided into three classes: (i) class I fusogens, which are dominated by α-helical coils; (ii) class II fusogens, which consist predominantly of β-sheets; and (iii) class III fusogens, which feature both secondary structure types.

**(B) Cell-cell fusion**: Some viruses such as vaccinia virus (VV) and herpes simplex virus (HSV) induce the expression of proteins on the surfaces of infected cells that attract uninfected cells and cause them to fuse with the infected cell at low pH values to form a multinuclear cell known as a syncytium [11, 13, 22, 23]. Syncytium formation represents a very efficient way for a virus to spread within a host: it circumvents the immune response and creates a good site of replication for a nuclear-replicating virus. It should be noted that syncytium formation is not always regarded as an entry mechanism per se.

**(C) Endocytosis:** Once the cell internalizes the virus, it is then delivered to an acidic pit, a so-called early endosome. The virus then may be transferred into a late endosome and then to a lysosome. Alternatively, due to the low pH value in the lumen of endosomes, the viral membrane can fuse with the endosomal membrane, releasing the viral genome into the cytoplasm [12]. After exiting from endosomes, some adenoviruses or poxviruses may use microtubules for transport within the cytoplasm. Once in cytoplasm, some viruses move toward the nucleus to deliver their cargo inside the nucleus, whereas the NCLDVs usually remain in cytoplasm to initiate their replication cycle. Dynamin GTPase may have a key role in regulating most endocytic pathways. During virus entry, dynamin is deposited in the neck of the endocytic pit toward the cytoplasm leading to the excision of the pit [24, 25]. There are several major endocytosis-based pathways that viruses can use to enter cells and evade the host’s immune system. These pathways differ in terms of the types of particles involved and the molecules that are important in the process. The most important viral entry pathways are as follows:

1. **Phagocytosis** (cell eating), which occurs in specialized mammalian cells (so-called professional phagocytes, e.g., dendritic cells and macrophages) that engulf large and essential particles. Viral entry by this pathway typically involves the formation of large extracellular projections, and the internalized virus is taken into a phagosome. Actin and RhoA are typically necessary for this process.
2. **Pinocytosis** (cell drinking), which is the process by which cells take up solutes and fluids. Pinocytotic processes can be further classified based on the membrane structures and types of molecules they are associated with. Macropinocytosis is a nonspecific process, and particles internalized by this route may not be essential for the cell. When it is exploited by viruses, interactions between viral proteins and cell receptors activate intracellular signaling and actin rearrangements that form ruffles or filopodia on the external surface of the host cell. The ruffles then close up to form a vesicle known as a macropinosome, which carries the virus into the cytosol. Actin, Rho GTPases (Rac and Cdc42), PI3K, and Na+/H+ exchange are usually required for this pathway, and kinases are required to regulate macropinosome formation and closure. Although dynamin might not be required for some viruses to enter via macropinocytosis, some strains of adenoviruses and poxviruses require dynamin to enter the cell.
3. **Clathrin-mediated endocytosis**, which is the process by which the cell internalizes the virus in a clathrin-rich flask-shaped invagination/cavity (vesicle) known as a clathrin-coated pit. The virus is then delivered into the cytoplasm via endosomes. Clathrin and cholesterol are required, and dynamin and transferrin are usually involved in pit formation.
4. **Caveolar/raft endocytosis**, which is similar to clathrin-mediated endocytosis but involves pits containing caveolin-1 rather than clathrin. The internalized virus is delivered to the cytoplasm in cave-like bodies known as caveolae or caveosomes, whose internal pH is neutral.
5. **Endocytosis based on other routes.** These pathways involve vesicles that contain neither clathrin nor caveolin. However, like the clathrin- and caveolin-based pathways, they generally require dynamin, cholesterol and/or lipids. Interestingly, lymphocytic choriomeningitis virus uses a dynamin-, clathrin-, and caveolin-independent route that is also independent of actin, lipid rafts, and the pH [26, 27].

## Mechanisms of attachment and entry utilized by large and giant DNA viruses

Members of all ten viral families covered in the review infect a wide range of potential hosts, including humans, other mammals, invertebrates, fish, protozoa, and algae, causing serious problems in public health, livestock farming, and aquaculture (Table 1). As suggested by this diversity of potential hosts, they can use many different mechanisms to enter host cells, and members of the same viral family may use very different mechanisms to enter a given host cell type. To ensure an efficient virus infection, a virus may utilize more than one mechanism to enter a given host cell.

**Table 1:** Entry mechanisms utilized by large and giant DNA viruses. I, linear dsDNA; O, circular dsDNA; N, nuclear replication; M, cytoplasmic replication; E, enveloped; D, non-enveloped; S, icosahedral virus

Features/replication	Genus or subgroup	Host	Topology	Entry
Adenoviridae: N; I; S; E; ~70–90 nm	Mastadenovirus	Mammals	S; D; contains long or short fibers	Endocytosis or macropinocytosis
	Aviadenovirus	Birds		
	Atadenovirus	Birds, ruminants, squamata, marsupial		
	Siadenovirus	Frog, birds, turtle		
	Ichtadenovirus	Fish		
Ascoviridae: N; O; 130 diameter × 200–400 nm length	*Diadromus* spp., *Heliothis* spp, *Spodoptera* spp. *Trichoplusia* spp.	Insects	E; no protrusions	-
Asfarviridae: M and N; I; E; 175–215 nm	African swine fever virus	Swine	E; short protrusions	Endocytosis or macropinocytosis
Baculoviridae: E; N; O; E; the nucleocapsid is ~21 × 260 nm	*Alphabaculovirus*	Lepidopteran	E; gp64 at surface	Fusion or endocytosis
	*Betabaculovirus*	Lepidopteran-specific		
	*Gammabaculovirus*	Hymenopteran-specific		
	*Deltabaculovirus*	*Culex nigripalpus*		
Herpesviridae: N; I; E; 150–200 nm	*Alphaherpesvirinae* (5 Genera)	Human or vertebrates (mammals, birds, fish, reptiles, and amphibians)	S; E; short protrusions (short envelope protein and phage-like tail)	Fusion, endocytosis or macropinocytosis
	*Betaherpesvirinae* (4 Genera)			
	*Gammaherpesvirinae* (4 Genera)			
	*Ictalurivirus*	Fish		
Iridoviridae: M; I; E and D; 120–350 nm	*Ranavirus*	Amphibians, reptiles	S; E and D; short surface protein	Fusion or endocytosis
	*Megalocytivirus*	Fish		
	*Lymphocystivirus*	Fish		
	*Iridovirus*	Crustaceans, insects		
	*Chloriridovirus*	Mosquitos		
Mimiviridae / Marseilleviridae: M; O / I; D; 200–600 nm	Mimivirus, Mamavirus, Megavirus, Moumouvirus, etc.	Mostly Protozoa; many viruses are isolated from environmental samples and the original host is unknown.	S; D; Long fibers, Marseilleviruses usually harbor short or no fibers	Phagocytosis-like
	Marseillevirus, Lausannevirus, etc.			
Phycodnaviridae: N; I; E; 100–220 nm	*Chlorovirus*, *Prasinovirus*, *Prymnesiovirus* and *Phaeovirus*	Marine protozoa and Algae	S; E; no fiber	Cell wall degradation or fusion
Poxviridae: M; I; E; 220–450 nm long and 140–260 nm wide	Orthopoxvirus	Human, primates, camels, rodent	E; short surface proteins	Fusion or macropinocytosis
	Leporipoxvirus	Rabbit		
	Squirrelpox virus species	Squirrel		
	Crocodylidpoxvirus	Nile crocodile		
	Molluscipoxvirus	Immunosuppressed human		
	Parapoxvirus	Superorder *Laurasiatheria*		
	Yatapoxvirus	Primate		
	Suipoxvirus	Swine		
	Cervidpoxvirus	Deer		

### *Adenoviridae*

Adenoviruses (Ad) are non-enveloped icosahedral viruses with diameters of 70-90 nm (Fig. 1) that can be divided into seven groups and 50+ serotypes. They harbor 30 to 40-kb linear dsDNA genomes encoding around 45 proteins, and they replicate in the nucleus. Their genomes encode fiber proteins with a conserved N-terminal tail, a shaft, and a globular knob domain. The lengths of these fibers are similar within a serotype, but Ad-F and Ad-G encode two fiber proteins: short and long [28, 29]. The fibers bind to a wide range of cell receptors [30]; upon binding at the plasma membrane, the fibers become detached from the viral core and remain at the surface, while the core enters the cell [30–32]. The coxsackie-adenovirus receptor (CAR) is a functional receptor for most Ad strains [33]; it is expressed in the tight junctions in the epithelial cells of some human tissues (brain, heart and pancreas) and various tumor cells, but not in mice or primates [34, 35] (Table 2). The long viral fibers are flexible enough to permit the fiber knob to interact with CAR, bringing the penton base of the viral capsid into contact with integrins in the host cell membrane. Other cellular receptors targeted by adenoviruses include CD46, CD80, CD86, desmoglein-2, heparan sulphate, sialic acid, major histocompatibility complex-1-α2, and vascular cell adhesion molecule-1. Ad-2, Ad-5 and egg drop syndrome virus enter host cells via clathrin-mediated endocytosis [36–38], whereas Ad-3, Ad-5 and Ad-35 enter via macropinocytosis [37, 39]. Longer lists of cellular receptors and entry pathways exploited by adenoviruses are given in Tables 2 and 3.

**Table 2:** Attachment cellular receptors used by adenoviruses

Cellular receptor	Adenovirus strains
CAR	Ad-12, 31, 2, 5, 9, 19a, 19p, 4 and 41; but not Ad-3, 7, 21, 11, 14, 35 nor 30; due to the structure conformation of the fiber protein [34, 40–42]
CD46	Ad-16, 21, 50, 11, 14, 34, 35, 19a and 37; but not Ad-3 or 7 [40–46]
CD80 & 86	Ad-3 and 7 [47]
DSG2	Ad-3, 7, 11 and 14 (only human DSG, but not mouse homolog) [48]
HSPG	Ad-2 and 5; but not Ad-35 [49]
Integrins	Ad-3, 35, 2, 5 and D60 through YGD motif instead of RGD [50]
MHC1-α2	Ad-5 utilizes α2 domain of MHC-I (MHC-I-α2) [51]
Sialic acid	Ad-8, 19a and 37; but not Ad-9 or 19p [52]
VCAM-1	Ad-5 [53]
GD1a glycan	Ad-8, 19a and 37; but not Ad-5, 9 or 19p [54]

### *Herpesviridae* (order *Herpesvirales*)

Herpesviruses (HVs) have an enveloped icosahedral virion (150-200 nm) containing a 120 to 240-kb linear dsDNA genome encoding 100-200 proteins (see Figure 1 and Table 1). They replicate in the nucleus. The >70 known members of this family include eight human pathogens: HSV-1, HSV-2, CMV, EBV, KSHV, VZV, HHV-6 and HHV-7. HVs are rich in glycoproteins (GPs) that can form heterodimeric complexes to facilitate attachment and entry [55, 56]. Several proteins are involved in their attachment, including viral GPs (gB, gC, gD, gH/gL, and the gH/gL/gO complex) and host cell proteins such as HVEM, integrins, heparan sulphate, syndecan, and neuropilin [57–62]. HVEM was the first recognized receptor for HSV-1/2 gD (see Table 3). HV has a bacteriophage-like short tail whose role in entry is currently unknown [63]. Interestingly, an analysis of cytomegalovirus (CMV) showed that the genomes of clinical samples contain at least 19 genes that are absent in laboratory-acclimated strains [64]. Three of these missing proteins, UL128, UL130 and UL131, contribute to viral entry by binding to gH/gL [64–69]. HVs generally enter host cells by endocytosis or fusion with the plasma membrane [149, 71–77]. HSV-1, CMV, EBV, KSHV and VZV enter via endocytosis [78, 61, 79–87]. KSHV has been observed to enter endothelial cells by pinocytosis [88] but enters monocytes via some other mechanism that may involve heparan sulphate, integrins, and the induction of Src and PI3 K signaling [89]. Details on the entry mechanisms of HVs and receptors mediating their attachment and entry can be found in Table 3.

**Table 3:** Entry mechanism and/or cellular receptors used by viruses. The cell types used in entry assay are mentioned whenever possible; otherwise, multiple cells might be used. “∞” means “interacts with”

Virus	Cells	Entry method and/or attachment receptors
Adenoviruses		
Ad-2/5	–	Clathrin, myeloid, and αvβ3- and αvβ5-integrins-mediated endocytosis [36, 90]
Ad-2	–	Macropinocytosis [90]
Ad-5	Afferent lymph DCs	Actin-dependent macropinocytosis [39]
Ad-3/35	EpC and haematopoietic	PI3K, Rho GTPases and dynamin-dependent macropinocytosis [37, 91]
Egg drop syndrome virus	Duck embryonic FbCs	Low pH, clathrin-mediated endocytosis [38]
Herpesviruses		
HSV-1	HeLa, CHO and keratinocytes, but not neuroblastoma	Low-pH endocytosis [92–94]
	Vero cells	Fusion [92]
	CHO	Viral gB and gD, and cellular Nectin-1, HVEM and PILR-α are required for infection; gD ∞ Nectin-1 and gB ∞ PILR-α [95]; gD ∞ Nectin-1 and gB ∞ PILR-α [96–99]
	EpC, neuron and keratinocytes	gH/gL (RGD motif) ∞ αvβ6- and αvβ8-integrins [100]; gH/gL binds to αvβ3-integrin activating IFN-I and NF-κB [101]
	CHO, HeLa, Vero	gD ∞ HVEM [102, 103]
	HeLa	Syndecan-1 and syndecan-2 [104]
	Nectin-1 or HVEM-deficient murine dermal FbCs	Delayed virus entry; HS could be an alternative receptorl; dynamin and cholesterol could be involved [105]
	Murine cornea	HVEM and nectin-1 are crucial for infection [106]
	Human oligodendrocytic cells	Proteolipid protein is required in entry [107]
	–	gD triggers fusion by forming complexes with gB or gH/gL [108]; gB ∞ non-muscle myosin heavy chain IIA [109]
	CHO and fibroblasts	gC, gB and gD are required for entry [110]; gD ∞ 3-O-sulfated HS [111]
HSV-2	Retinal EpCs	Nectin-1, HVEM and PILR-α [95]; gD ∞ Nectin-1 and PILR-α and gB ∞ PILR-α [96–99]
HSV-6	–	gH/gL/gQ ∞ CD46 [112–114]; gB and the gH/gL/gQ complex are required for cell-cell fusion [115]
HSV-7	CHO	gB ∞ HS [116]
CMV	Fibroblast, EnC and retinal EpC	Fusion or endocytosis [78, 117]
	Multiple cells, e.g. CHO, myeloid, EpC, EnC and FbC	gB ∞ epidermal growth factor receptor [118] or integrins (does not depend on RGD motif) [119]; gH ∞ αvβ3 integrin as a co-receptor [120]; gB or gH/gL are required for syncytium [121, 122]. gH/gL/UL128/130/131 and gH/gL/gO complexes are essential for fusion [123]
EBV	B lymphocytes	Endocytosis [124, 125]; gp350/220 ∞ complement receptor 2 (CR2, CD21) [126, 127]. gH/gL (KGD motif) ∞ αvβ6- and αvβ8-integrins [128, 79]; gp42 ∞ HLA to induce membrane fusion through gH/gL and gB [, 80, 81, 110, 126].
	EpCs	Fusion [125]; macropinocytosis and lipid raft-dependent endocytosis [82]
	B cells, but not EpC	gp42/gH/gL complex mediates fusion [83]
	Nasopharyngeal EpC	gB ∞ Neuropilin-1 [82]
	Polarized cells	BMRF2 protein ∞ integrins [84]
KSHV (HHV-8)	EnC and FbC	DC-SIGN, pH and clathrin mediated endocytosis [85–87]
	Endothelial cells	Macropinocytosis [88]
	Monocytic THP-1 cells	Endocytosis; clathrin, caveolin, HS, DC-SIGN, integrins, NF-κB, Src, and PI3K signaling are involved [89].
	Human dermal microvascular EnC	gB ∞ ESCRT-0 component Hrs (hepatocyte growth factor-regulated tyrosine kinase substrate) promoting macropinocytosis [129]
	–	gB (RGD motif) ∞ integrins [130, 57]. gB, gH/gL and K8.1 ∞ HSPG induces fusion [58, 59, 60].
VZV	B cells	Endocytosis [61]
	VZV-permissive human melanoma cells expressing integrins	gB and gH-gL ∞ αV integrins [62]
Ovine herpesvirus 2	–	gB and gH/gL induce cell-cell fusion [131]
Poxviruses		
VV MV / EV	HeLa	Low-pH, dynamin, actin, and cholesterol-dependent macropinocytosis [132–137]
VV MV	HeLa cells	Bind to CD98 and enters via endocytosis [138]
VV MV / EV	DCs	Dynamin and pH-independent macropinocytosis [139], cholesterol (lipid raft), PS, actin, kinases, GTPases, integrins and Na+/H+ exchangers are required [134, 140, 141].
VV-MV	HeLa or A549	Low-pH, and serine/threonine kinase PAK1 and tyrosine kinase [142].
VV	Human pancreatic carcinoma cell lines	Entry enhanced by vascular endothelial growth factor A and Akt signaling pathway [143].
VV	Leukocytes	Attach to heparin and laminin [144, 145]
VV	Fibroblast or HeLa	Tumor necrosis factor receptor associated factor 2 [146]
VV	*Drosophila* DL1 cells	Macropinocytosis [147]
VV	*Drosophila* S2 cells	Low-pH endocytic pathway that requires EFC proteins [148]
Myxoma virus	Leukocytes	Attach to heparin [144]
VV and myxoma virus	FbCs BSC-40	Inhibition of HS affects entry, but laminin blocks binding of VV [144].
WR and IHD-J	HeLa	PS, kinases and actin macropinocytosis; IHD-J MV induces filopodia; WR utilizes tyrosine kinase, PI3K and Rac1 to activate blebs [136].
	HeLa, B78H1 and L cells	Inhibited by soluble heparin [149, 150]
	B78H1 and BSC-1	Require endosomal acidification [149, 150]
WR, monkeypox virus and cowpox virus	–	Low-pH [150, 151]
IHD-J, Copenhagen and Elstree strains	–	A pH-independent fusion [150, 151]
WR EV	–	Gas6 protein enhances entry by bridging viral PS to TAM (Tyro3/Axl/Mer) receptor tyrosine kinases [152].
EVs	–	Expression of A33 and A36 at plasma membrane of the infected cells mediates the repulsion between EVs toward uninfected cells leading to rapid spread of virus [153]. A56 (hemagglutinin) interact with K2 (serine proteinase inhibitors) forming A56-K2 complex that co-localizes at the cell surface blocking the superinfection and fusion [154–157]. A56-K2 complex interacts with A16 and G9 subunits and prevents the superinfection [158].
Iridoviruses		
Tiger frog virus, *Ranavirus* genus	HepG2 cells	pH, cholesterol, dynamin, actin and caveolin-mediated endocytosis [159]
Frog virus 3, *Ranavirus* genus	BHK-21 cells	Low pH and clathrin-mediated endocytosis [160]
ISKNV, *Megalocytivirus*	Mandarin fish fry cells	Major capsid protein ∞ caveolin-1 and induces caveolin-endocytosis [161, 162]
SGIV	Grouper spleen cell line	pH-dependent clathrin-endocytosis and macropinocytosis [163]; the deletion of VP088 envelope protein inhibits viral entry [164].
Large yellow croaker iridovirus	Bluegill fry (BF-2) cells	037L (RGD motif) ∞ integrins inducing fusion [165, 166]

### *Baculoviridae*

Baculoviruses are arthropod-specific enveloped virus with nucleocapsid dimensions of 21 × 260 nm (Fig. 1). They have circular dsDNA genomes of 80-180 kb that encode 100-180 proteins and replicate in the nucleus. They are used in biocontrol against insects, and as vectors for gene transfer and protein expression. Consequently, their entry into insect, human, and cancer cells has an increasing biological impact (see Tables 1 and 3). Two baculovirus phenotypes have been characterized: budded and occlusion-derived. Viruses of this family express two crucial fusogens, gp64 (class III) and F (class I), which are functionally analogous and can both trigger low-pH membrane fusion during endocytosis. There are evidences that gp64 facilitate virus entry and fusion with the plasma membrane [167–170]. Bombyx mori nucleopolyhedrovirus (BmNPV) enters *Bombyx mori* (BmN) cells via cholesterol-dependent macropinocytosis [171], while Autographa californica multiple nucleopolyhedrovirus (AcMNPV) grown in *Spodoptera frugiperda* (sf9) cells enters human hepatocarcinoma (HepG2) and embryonic kidney (293) cell lines via a dynamin-, raft- and RhoA-dependent phagocytosis-like mechanism [172], but clathrin-mediated endocytosis or macropinocytosis may not be involved in the virus uptake. However, recombinant AcMNPV from sf21 cells enters BHK-21 cells via low-pH clathrin-mediated endocytosis [173]. Additionally, a pseudotyped vesicular stomatitis virus (VSV) encoding gp64 grown in Sf9 cells enters the Huh7 and 293 cells via macropinocytosis and endocytosis, which is mediated by viral gp64, and cellular cholesterol, dynamin and clathrin [169]. This process also requires the host cell proteins HSPG and syndecan-1 [174], as well as cholesterol [169, 175].

### *Poxviridae*

Poxviruses are widely distributed enveloped viruses (∼360 × 270 × 250 nm) that replicate in the cytoplasm (Fig. 1) [176]. They harbor a 130 to 375-kb linear genome that encodes ~200 proteins. Vaccinia virus (VV) is a prototypic virus of this class that was used as a smallpox vaccine. It exists in three forms [177, 178]. The first is the mature virion (MVs, also known as the intracellular mature virus, IMV or INV), which has a brick-shaped structure; it is the most abundant, stable and simple form and is active in host-host transmission. The second form is the wrapped virion (WV or intracellular enveloped virus, IEV), which contains an MV core wrapped in two membranes. WVs travel to the cell periphery via microtubules and fuse with the plasma membrane, and they are then released by exocytosis as the third form, the extracellular virion (EV, or cell-associated extracellular enveloped virus, CEV, or extracellular enveloped virus, EEV), which is specialized for exiting and cell-to-cell transmission within the host.

Four proteins are used for attachment to the cell surface (A26, A27, D8 and H3), and the MV displays the so-called entry-fusion complex (EFC), which consists of 11 proteins (A16L, A21L, A28L, F9, G3L, G9R, H2, J5, L1R, L5R and O3L). These proteins interact with one another and mediate virus-cell fusion, membrane disruption, and cell-to-cell fusion [176, 179, 180] (Tables 3 and 4). Inhibition of any of these proteins destabilizes the complex and hence perturbs viral entry. MV enters host cells via endocytosis or fusion with the plasma membrane, leaving the virus in endosomes [179–184] (see Table 3). Notably, the mechanisms of fusion for MVs and EVs at the plasma membrane and endosome are identical, and both require EFC proteins. VV (MV/EV), WR, and IHD-J enter HeLa cells via macropinocytosis [132, 134–139] and have also been suggested to enter via a parallel endocytotic mechanism [138]. In *Drosophila*, VV enters DL1 cells by macropinocytosis [147], but it enters S2 cells via endocytosis [148].

**Table 4:** Poxviruses entry proteins, cellular receptors and functions. 1, N-terminal, 2, C-terminal transmembrane domain

Protein	Roles
**Attachment**	
A26	Binds to laminin [185]; A25 and A26 may act as fusion suppressors [180, 186].
A27	Binds to heparan sulfate, but not chondroitin [187]; binds with A17 protein forming a complex that mediates pH-dependent cell-to-cell fusion and syncytium formation [188].
D8	Binds to chondroitin sulfate and mediates the adsorption of MV [189]
H3	Binds to heparan sulfate [190]
**Entry (entry-fusion complex, EFC)**	
A16L	2; interacts with G9 and with A56/K2 to prevent superinfection; A16-deficient virion fails to induce syncytia [191].
A21L	1; interacts with H2; [192]
A28L	1; interacts with H2 and both are required for entry and cell-cell fusion [177, 179, 193–195].
F9	2; important for entry; F9-deficient virus binds to the cell, but the core fails to penetrate into the inside [177, 196].
G3L	1 [197]
G9R	2; binds to A16 and A26 suppressing fusion [198, 199].
H2	1, binds to A28 and both are required for entry and cell-cell fusion [177, 179, 194, 195].
J5	2 [200]
L1R	2; binds with uninfected cell receptors; L1 mutant virus is lethal, as it is required in assembly and fusion [177, 196, 201]
L5R	2 [202]
O3L	1 [203]

## Giant viruses (*Mimiviridae* and *Marseilleviridae*)

These families comprise the largest known viruses, so-called giant viruses (GVs). They have genomes of ~0.5-2.5 Mb that encode 400-2500 proteins, and they replicate in the cytoplasm. Representatives of these families have been isolated from diverse habitats, including bronchoalveolar lavage fluid [204] and stools [205] from patients with pneumonia, insects [206], and leeches [207] (for a detailed review, see reference [208], [209]). The nature of the relationship between giant viruses and pneumonia remains to be elucidated [209–212]. Briefly, the giant viruses were detected by serological and genomic methods in patients with respiratory symptoms. Moreover, recent images show giant virus- and virus factory-like structures in number of human cells [213].

**Fig. 3:**
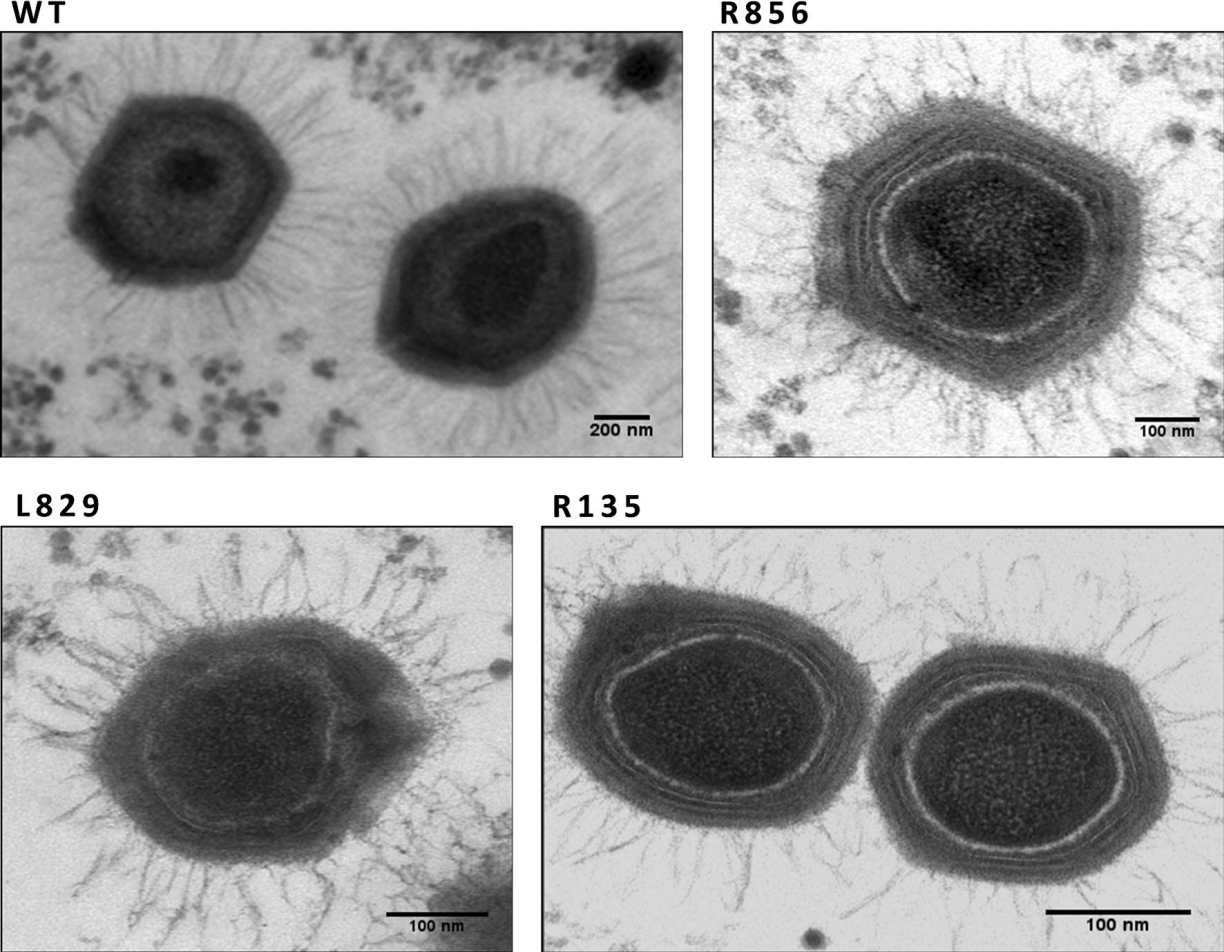
Silencing any one of the four fiber-associated proteins in mimivirus produces viruses bearing short and deformed fibers compared to the wild-type control (WT). The images are adapted from reference [216]

Mimivirus virions are 500 nm in diameter, with a 1 Mb dsDNA genome encoding 900 proteins. Their surfaces are completely covered with fibers (120 nm long) attached to the capsid via a disc-shaped feature except at one capsid vertex (Fig. 1). The outer fibers may play some role in the virus’ attachment to or entry into host cells [214, 215], but the details of its mechanisms of attachment and entry are unknown. Proteomic and gene silencing experiments revealed that the fibres consist of at least four proteins (R135, L725, L829, and R856); viruses in which any of these proteins are silenced exhibit short and deformed fibers [214, 216–219], as shown in Figure 3. Further structural analysis showed that R135 is a component of the fibers and is required for host cell entry [219]. In addition, a electron microscopy showed that L725 aggregates form fibre-like architectures [217]. The fibers’ shape differs from that in other viruses, and the fiber proteins exhibit no sequence similarity to proteins encoded by other viruses. It should be noted that some giant viruses lack external fibers – for instance, marseilleviruses (which are 200 nm in diameter with 350-kb circular dsDNA genomes) have topologies similar to those of mimiviruses but have only short (12 nm) or no fibers [216].

Mimiviruses enter amoebae or macrophages via a phagocytosis-like mechanism that depends on dynamin, actin and PI3-K [220, 221]. Unlike poxviruses, the entire virion with fiber can be seen inside the host. Further analyses showed that individual Marseillevirus virions enter *A. castellanii* cells via phagocytosis or in vesicles, endocytosis and micropinocytosis, were also suggested, but remain to be investigated [222]. Because the closely related Mimiviruses enter cells via phagocytosis, it seems very plausible that Marseillevirus could also enter via such a mechanism. It should be noted that the original host of most giant virus strains, including APMV, is not known; neither amoebae nor macrophages are their natural hosts. The tropism of these viruses and their interactions with their natural host cells thus remain to be elucidated.

### *Phycodnaviridae*

The *Phycodnaviridae* are marine enveloped viruses with dimensions of 100-220 nm that have 330 to 560-kb linear dsDNA genomes and replicate in the cytoplasm of algae (Fig. 1). Despite having algal hosts, their entry pathways resemble those used by bacteriophages and animal viruses. *Paramecium bursaria* chlorella virus (PBCV-1) attaches to host cells via a viral vertex and degrades the host cell wall at the site of attachment like a bacteriophage [223]. To this end, it encodes chitinases, chitosanase, β -1,3-glucanase, and alginase enzymes that catalyze cell wall lysis [224]; it also encodes potassium ion channel proteins, which have a putative role in entry [225, 226]. After entry, PBCV leaves an empty shell at the cell surface. Another member of this family, *Emiliania huxleyi* virus 86, enters host cells via endocytosis or fusion of the outer lipid membrane surrounding the capsid, which is similar to animal virus entry [227]. The intact virion can be seen in the cytoplasm before the capsid breaks down to release the genome. *Ectocarpus fasciculatus* virus infects zoospores or gametes of brown algae that lack cell walls [228]. It fuses with the outer plasma membrane of the host cell, leaving the capsid outside the cell surface, and injects its genomic cargo into the cytoplasm.

### *Asfarviridae*

These are enveloped viruses (175-215 nm, see Figure 1) with 170 to 190-kb linear dsDNA genomes encoding around 150 genes. They infect macrophages and monocytes of pigs and argasid ticks, and they replicate in the nucleus and/or cytoplasm. The early steps in the binding and entry of African swine fever virus (ASFV) into host cells are largely unknown [229]. The ASFV-E70 and Ba71V strains enter Vero cells and macrophages by low-pH-, dynamin-, and clathrin-dependent endocytosis, which requires actin, small GTPase Rab7 and PI3-K. Additionally, cholesterol may be needed to liberate the virus from endosomes into the cytoplasm [230–234]. There is also evidence that ASFV can enter via macropinocytosis, which requires actin, kinases and Na+/H+ exchange [235].

### *Iridoviridae*

The iridoviruses include both enveloped and non-enveloped viruses with dimensions of 120-350 nm that replicate in the cytoplasm of insect and fish cells (Fig. 1). They harbor 100 to 200-kb linear dsDNA genomes with circularly permuted and redundant termini. The enveloped viruses fuse with the cell membrane of the host cell, whereas the non-enveloped viruses enter via endocytic pathways [236] (see Table 3). Frog virus 3, tiger frog virus, and infectious spleen and kidney necrosis virus enter BHK-21, HepG2 and Mandarin fish fry cells, respectively, by endocytosis [159–162]. The VP088 protein encoded by SGIV facilitates both endocytosis and macropinocytosis into a grouper spleen cell line [163, 164].

### *Ascoviridae*

These viruses (~130 nm diameter, 200-400 nm in length) infect invertebrates; they replicate in the nucleus and harbor 150 to 190-kb circular dsDNA genomes that encode 180 proteins (Fig. 1). They are phylogenetically related to iridoviruses, and their entry mechanisms are obscure. However, Heliothis virescens ascovirus-3e infections are known to require actin rearrangement [237].

## Conclusion and future perspectives

Viruses enter host cells via several mechanisms, depending on the host cell type and viral strain. Concerns about the risks of viral outbreaks have prompted efforts to characterize emerging pathogens and predict the emergence and properties of new viruses. A further motivating factor for such studies is the possibility of developing non-cytotoxic antiviral drugs that act outside host cells by preventing viral attachment or entry rather than disrupting viral replication inside cells. This review details the entry pathways exploited by large dsDNA viruses. Their entry pathways are affected by several factors, including the external topology of the virions (particularly the presence of surface protrusions and their topology), the targeted cell type, the cellular receptors that are present, and the viral protein content.

While viruses from the same viral family often have similar topologies and encode proteins with similar sequences and structures, they may still use different entry mechanisms. As mentioned in Table 3, the virus protein(s) may bind to one or more receptors and co-receptors (see herpesviruses for examples). The binding may activate number of factors (proteins/pathways) that are relevant to infection. These factors could be characteristics of other entry pathways (see, for example, entry of KSHV). Additionally, the MV form of vaccinia virus can enter cells by direct fusion with either the plasma membrane or the membrane of a vesicle after endocytosis.

It is worth emphasizing that additional factors could affect the entry mechanism. Among these factors is protein sequence similarity; some viral proteins exhibit functional and structural similarities despite having little or no sequence similarity. For example, the HSV-1 protein gB is a class III fusogen that resembles (especially in its post-fusion conformation) the gG protein of the RNA rhabdovirus VSV and the baculovirus protein gp64 [72, 238–241]. Additionally, the EBV protein gp42 is a functional homolog of HSV gD, but the two share no sequence similarity [110]. The functional motifs of viral proteins appear to play central roles in determining the entry pathways available to specific viruses, so their analysis could enable prediction of entry pathways and virus-host cell interactions [14, 242]. Closely related viruses that infect the same host generally have similar functional motif profiles [242]. Another factor that may be important is ubiquitination of viral proteins inside host cells, which can affect infection and microtubule trafficking. For instance, the adenovirus protein VI recruits Nedd4 E3 ubiquitin ligases via interactions involving its PPxY motif [14, 61, 243, 244]. Biophysical factors may also affect viral entry. For example, the entry of CMV into vascular endothelial cells is promoted by low levels of shear stress [245]. Similarly, the fusion of the enveloped HSV requires a negative curvature of the lipid bilayer and can thus be suppressed by factors that prevent the formation of such negative curvature [246].

Differences in observed entry pathways for different strains or different samples of the same viral strain may be due to differences in experimental design and conditions [61], the use of a non-physiological host *in vitro* (e.g., non-wild-type cells), or the use of a laboratory strain whose gene content differs from that of the wild-type virus, as in the case of CMV [64]. It is generally accepted that cell lines (i.e., immortalized cells) often differ genetically and phenotypically from cells in native tissues (or primary cells). Consequently, the type of cell used when studying viral entry may profoundly affect the results obtained. It has also been shown that baculoviruses grown in different insect cell types enter mammalian cells via different mechanisms [247]. These results clearly show that there are several aspects of viral entry into host cells that are very poorly understood. Comparative studies could potentially shed important light on this topic and help to clarify unknown aspects of virus-host cell interactions. In addition, more comprehensive information on viral topology and protein sequences will help to understand virus tropism. Further studies in this area should focus on predicting viral entry mechanisms and the evolution of interactions between host cells and viruses. Efforts should also be made to identify optimal experimental conditions for viral entry in different cell types and for different viral families.

## Abbreviations

dsDNA: double-stranded DNA
CHO: Chinese hamster ovary
DC: dendritic cell
EnC: endothelial cell
EpC: epithelial cell
FbC: fibroblast cell
VV: vaccinia virus
WR: VV Western Reserve
IHD-J: International Health Department-J
AcMNPV: Autographa californica multiple nucleopolyhedrovirus
BmNPV: Bombyx mori nucleopolyhedrovirus
ISKNV: infectious spleen and kidney necrosis virus
SGIV: Singapore grouper iridovirus
ASFV: African swine fever virus
HHV: Human herpesvirus
HSV: herpes simplex virus type 1 or 2 (HSV-1 or HSV-2, also known as HHV-1 and HHV-2, respectively)
VZV: varicella-zoster virus (or HHV-3)
EBV: Epstein-Barr virus (or HHV-4)
CMV: cytomegalovirus or human CMV (HCMV or HHV-5)
KSHV: Kaposi’s sarcoma-associated HV (HHV-8)
VSV: vesicular stomatitis virus
CAR: coxsackie-adenovirus receptor
DSG2: desmoglein-2
ESCRT: endosomal sorting complexes required for transport
GAGs: glycosaminoglycan
GD1a: disialogangliotetraosylceramide
HLA: human leukocyte antigen
HS: heparan sulphate
HSPG: heparan sulphate proteoglycans
HVEM: herpesvirus entry mediator
IFN: interferon
MHC: major histocompatibility complex
PGs: proteoglycans
PI3-K: phosphatidylinositol 3-kinase
PILR-α: paired immunoglobulin-like receptor alpha
PS: phosphatidylserine
VCAM-1: vascular cell adhesion molecule 1
